# Food Niche Overlap of Avian Predators (Falconiformes, Strigiformes) in a Field and Forest Mosaic in Central Poland

**DOI:** 10.3390/ani11020479

**Published:** 2021-02-11

**Authors:** Jakub Gryz, Dagny Krauze-Gryz

**Affiliations:** 1 Department of Forest Ecology, Forest Research Institute, Sękocin Stary, Braci Leśnej 3, 05-090 Raszyn, Poland; 2 Department of Forest Zoology and Wildlife Management, Institute of Forest Sciences, Warsaw University of Life Sciences, Nowoursynowska 159, 02-776 Warszawa, Poland; dagny_krauze_gryz@sggw.edu.pl

**Keywords:** diet composition, food niche breadth, pellet analysis, breeding season, raptors, owls

## Abstract

**Simple Summary:**

Predators may present various feeding strategies, i.e., being either food specialists or opportunists. At the same time, their diets change to reflect the prey availability and to avoid competition for food resources. We performed this research in a highly transformed field and forest mosaic and in an area with a high abundance of avian predators (owls and birds of prey, ~133 breeding pairs in total). We calculated the food niche overlap statistics to show the competition for food resources between coexisting species. We assessed the diet composition on the basis of pellet analyses and the identification of prey remains collected from under nests during the breeding season. The food niches overlapped moderately with only one exception, i.e., the highest niche overlap was recorded for the common buzzard and common kestrel, two species preying in open spaces on field rodents but switching to soricomorphs when the former were scarce. On the contrary, the most separate food niche was that of the white-tailed eagle, which was the only species regularly preying on fish. Our results showed that the food niches of species coexisting in the same area were considerably separate, which is due to the fact that they prey on various prey species or search for them in different habitats.

**Abstract:**

Food niche overlap statistics are a common way to show competition for food resources in a group of animals. Niche breadths of various species are very variable and their diet composition changes reflecting prey availability. The aim of this study was to evidence the food niche overlap of the whole assemblage of avian predators (eight raptor and owl species, some of them reaching very high densities) in a field and forest mosaic of central Poland. The diet composition was assessed on the basis of pellet analyses and the identification of prey remains found under the nests in the breeding period. The extent of the niche overlap was calculated using a Pianka formula. The food niche overlap indices ranged from 0.02 to 0.93 (mostly below 0.5). The most separate food niche was that of the white-tailed eagle, who regularly preyed on fish. The highest niche overlap was recorded for the common buzzard and common kestrel, two species preying on field rodents, switching to soricomorphs when the former were scarce. Our results confirmed that the food niches of species coexisting in the same area were considerably separate, which is a result of preying on various prey species or searching for them in different habitats.

## 1. Introduction

Interspecific interactions among predators, including competition, shape ecological networks and food webs [1], and dominant predators strongly affect the distribution and abundance of submissive predators [2,3]. An example of this was found in an owl community in which the distribution of the dominant Ural owl *Strix uralensis* affected the spatial distribution of the smaller and competitively weaker tawny owl *Strix aluco* [2,4]. Competition will rise when food resources are limited and changes in the prey abundance on the short- [5] or long-term [6] can strongly influence the degree of food niche overlap.

In the case of many raptor species, the abundance of small rodents in particular plays an essential role. As an example, a long-term study conducted in central Poland showed that, in the years with low field vole *Microtus arvalis* abundance, the competition and food niche overlap between the common buzzard *Buteo buteo* and the Northern goshawk *Accipiter gentilis* rose strongly. When the abundance of rodents was high, the common buzzard preyed mainly on rodents, switching to birds (the main prey of the Northern goshawk) when the former were scarce [7]. At the same time, raptors preying on the same food category can avoid competition by hunting in different habitats. For instance, Northern goshawk and white-tailed eagle *Haliaeetus albicilla* both prey on large birds; however, goshawks hunt mainly in forests and open fields, while white-tailed eagles hunt mainly in the vicinity of water bodies [8,9].

Food niche overlap statistics are a common way to show competition for food resources in a group of animals that coexist in a given area [10,11,12]. At the same time, niche breadths in different populations of the same species can be highly variable, depending on the habitats they use [13]. In the case of the tawny owl, the food and environmental niche breadths were found to be highly correlated [14]. The overlap of the food niches of predators has been analyzed in few scientific papers. Most of them were related to carnivorous mammals and only a few dealt with more coexisting species [15,16,17,18,19,20]. A study from western Poland investigated the overlap of the food niches of the common buzzard and red fox *Vulpes vulpes* in an agricultural landscape [21]. Complex studies of the food niche overlaps of avian and mammalian predators were conducted for the winter period in the Bialowieża Primeval Forest [22,23]. Most studies involving birds of prey focused on only a few coexisting species [24,25,26,27,28,29]. Yet, in India, food niche overlaps for six different raptor species were analyzed [30]. To our knowledge, no study so far described the food niche overlap in a general assemblage of avian predators in human-modified landscape, i.e., field and forest mosaic.

In the light of this, the aim of this study was to evidence the food niche overlap within the general assemblage of avian predators (raptors and owls) in the breeding season in a field and forest mosaic of central Poland. For methodological and statistical reasons, eight (out of 12 recorded in the area) species of avian predators (some of them, e.g., the common buzzard, reaching very high density [6]) were included in this study. Considering the high density of raptors in the study area and the coexistence of the whole assemblage of avian predators (i.e., most of them bred in small woods), we expect that their food niche overlaps remain limited.

## 2. Materials and Methods

### 2.1. Study Area

**Figure 1:**
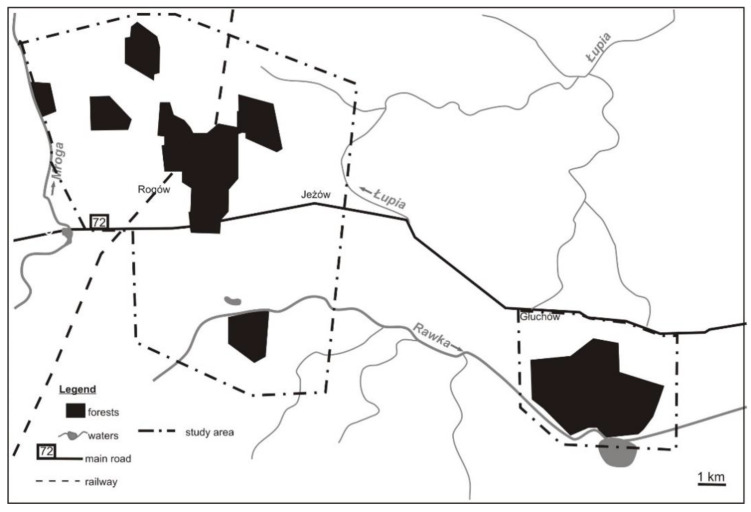
The distribution of forest complexes in the study area (central Poland, vicinity of Rogów village, Experimental Forest Station of Warsaw University of Life Sciences).

The study was performed in central Poland, in the area of the Experimental Forest Station of Warsaw University of Life Sciences, in the vicinity of the village of Rogów (51°49′17.98″ N, 19°53′54.15″ E). The study area comprised ~105 km^2^ of field and forest mosaic and was separated in two sub-areas. Forests accounted for around 25% of the area (~2400 ha) and formed seven complexes (70–1000 ha) (Figure 1). The remaining parts of the area were arable lands (59%), orchards (5%), grasslands (5%), and scattered buildings [31]. Two small rivers went through the study area, and 1 km^2^ of river ponds were located in the southern part. The main forest habitat types were fresh mixed forest and fresh broadleaved forest (together 83%). The main forest-forming species was Scots pine *Pinus sylvestris*.

Overall, the mean number of breeding pairs (as recorded on the basis of complex census of breeding pairs conducted in the whole study area every year [6,8,32,33,34,35,36,37]) of all twelve species of avian predators recorded annually was 133.2 and the density was 126.9 pairs/100 km^2^ (Table 1). The most numerous birds of prey were the common buzzards, whose density increased over the last decades [6]. As for owls, the most numerous were the tawny owls [32,33]. The sparrowhawk *Accipiter nissus* and long-eared owl *Asio otus* were the second most numerous diurnal and nocturnal avian predators [32,33,34]. In the case of other species, less than ten pairs were recorded annually (Table 1). In the last ten years, breeding of the barn owl *Tyto alba* and little owl *Athene noctua*, which were numerous in the 1970s, was not confirmed [32]. The osprey *Pandion haliaetus*, which nested in the study area in the beginning of 1960s, was also no longer recorded [35].

### 2.2. Material Collection and Analysis

We studied the food composition and food niche overlap of eight birds of prey and owls in seven to 16 breeding seasons, depending on the species (Table 2). For three species, we used published data: the common buzzard [6], Northern goshawk [8], and sparrowhawk [34]. Published data for the tawny owl [38] and long-eared owl [39,40] were supplemented with unpublished data from 2015–2018. For the diet composition of the white-tailed eagle, common kestrel *Falco tinunculus*, and Eurasian hobby *Falco subbuteo*, only original data were used (Table A1, Table A2 and Table A3).

Due to methodological reasons (low sample size of identified prey items or different food composition), data on four species occurring in the study area (Table 1) were not included. Pellets of the raven *Corvus corax* contained a high proportion of seeds and inorganic remains; therefore, different methods of food composition assessments were implemented for this species compared to other bird species [37], and food niche overlaps calculations would be questionable in this case. Due to the small sample size of identified prey items, the diets of the marsh harrier *Circus aeruginosus*, Montagu’s harrier *Circus pygargus*, and honey buzzard *Pernis apivorus* were also excluded from the analysis.

The diet composition of birds of all species included in this study was assessed on the basis of pellet analyses and the identification of prey remains. Each year, we searched the study area for new breeding sites, while known nests, nest boxes, and nest tree holes were checked. We also noted courtship displays, as well as the presence of single birds, to help to locate new breeding sites. Then, each known breeding site was visited every two weeks in the breeding season, i.e., from April to July to collect prey remains and pellets [6,34,38,39,40]. Such time interval limited disturbance caused by the researchers’ visits to the nest and ensured that the prey collection reflected possible changes in the prey composition over time. Each year we collected pellets and prey remains in the breeding sites of all known pairs (Table 1).

In the laboratory, we systematically assigned the prey remains with the aid of keys [41,42,43,44,45]. A collection of feathers and skulls was used for comparisons. In some cases, we performed histological analysis of hair [46]. We attempted to avoid double counting of prey, e.g., we included an avian prey assigned to the species level based on feathers from a plucking site; at the same time, bird remains from one pellet found in the same territory were excluded.

We presented the diet composition as a share of a given taxon in a total number of prey items. The food niche breadth was calculated according to Simpson formula (D = 1/∑p_i_^2^, p_i_ – % share of a given food category in a diet) for 11 food categories: soriciforma, forest rodents (*Apodemus flavicollis* and *Myodes glareolus*), field rodents (*Apodemus agrarius*, *A. sylvaticus*, *Microtus* spp., *Mus musculus*, and *Micromys minutus*), other mammals, small birds (up to 35 g), medium birds (36–399 g), big birds (400 g and heavier), fish, amphibians, and invertebrates. The body masses of certain species were based on the literature data [23,47]. The estimate of niche overlap between two species was calculated with a Pianka formula [48], and the results were within 0 (total niche separation) to 1 (full overlap of niches). We compared the similarity of the diet compositions using cluster analysis based on the unweighted pair-group average (UPGMA) and Morisita’s index of similarity. Cluster analyses were performed using the Past 4 program [49].

## 3. Results

**Figure 2:**
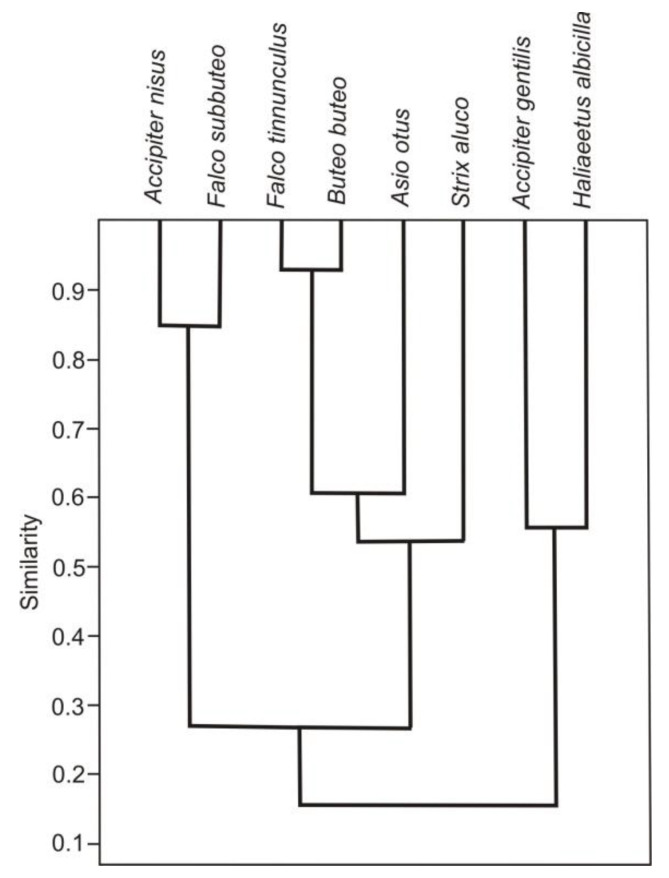
Cluster analysis based on the unweighted pair-group average (UPGMA) and Morisita’s Index of Similarity, which estimates the similarity in the diet composition between avian predators in the breeding season in the area of study (central Poland, vicinity of Rogów village, Experimental Forest Station of Warsaw University of Life Sciences), based on pellet analysis and prey remains collection.

The food niche overlap indices ranged from 0.02 to 0.93, however for most species were below 0.5 (Table 3). The most separate food niche was found for the white-tailed eagle (the mean niche overlap with all the other species was 0.16, min 0.02, max 0.56). The only species with whom its niche overlap was over 0.5 was the Northern goshawk (Table 3). Cluster analysis also showed the highest similarity between the diet compositions of those species, while their diets were very different from those of all other species under study (Figure 2).

The two species whose diets overlapped to the highest extent were the common buzzard (mean niche overlap 0.44, min 0.15, max 0.93) and common kestrel (mean 0.45, min 0.15, max 0.93), with the highest overlap values recorded for the common kestrel and common buzzard (0.93, Table 3) and the highest similarity index recorded in this case (Figure 2). The common buzzard and common kestrel were also the species with one of the highest niche breadths (5.2 and 4.4, respectively, Table 2). Approximately 30% of prey items caught by common buzzards and common kestrels were field rodents (Table 2). The other food component that appeared in high number in the diets of both species was soricomorphs.

The diets of the common buzzard and common kestrel overlapped extensively with that of the long-eared owl (Table 3); the diet of long-eared owl was also relatively similar to that of the common buzzard and common kestrel according to cluster analysis (Figure 2). Out of all species under study, the long-eared owl was the species that preyed most often on field rodents, i.e., rodents accounted for more than 80% of all consumed prey items (the long-eared owl also had the narrowest food niche of all the species in question, the value being 1.5). The food niche overlap between the diet composition of this owl and other avian predators was moderate (mean 0.28) (Table 2). Cluster analysis indicated a relatively high similarity index between the prey composition of the long-eared owl, common buzzard, common kestrel, and another owl species, the tawny owl (Figure 2). The tawny owl also preyed on field rodents, however less intensively than the long-eared owl, common buzzard, or common kestrel, and forest rodents were more important for the tawny owl (Table 2). The food niche breadth of the tawny owl reached the second highest value equaling 4.6 (Table 3).

A high similarity was also found for the diet composition of sparrowhawks and hobbies (Figure 2). The two species preyed on small birds (51% and almost 32% of all identified prey items, respectively, Table 2), and their diets overlapped considerably (the index value being 0.54, Table 3). Other diet components typical for hobbies were insects (which accounted for over 60% of all prey items) (Table 2 and Table A3). The diet of the sparrowhawk overlapped considerably also with that of the Northern goshawk (the index value being 0.54, Table 3). Medium-sized and big birds were most numerous in the diet of the Northern goshawk, while small birds were the most numerous in the diet of the sparrowhawk (Table 2).

## 4. Discussion

In this study, we evidenced the food niche overlap of the general assemblage of avian predators (raptors and owls, eight species in total) in the breeding season in a field and forest mosaic of central Poland. Because of the coexistence of the entire, very abundant, assemblage of avian predators, we expected that their food niche overlap remained limited. We assessed the food niche overlap indices between pairs of species, which ranged from below 0.1 to above 0.9. Nevertheless, for most pairs of species, the index was relatively low (i.e., below 0.5).

The most separate food niche was found for the white-tailed eagle, by far the largest avian predator in the study area, which preyed mainly on big birds and fish. Fish were absent from the diets of other species, while big birds were also preyed upon by the Northern goshawk, the species whose diet was the most similar to that of the white-tailed eagle. Hunting white-tailed eagles were observed mainly in the area of two fish farms located in the study area. The ponds are intensively stocked up with carps *Cyprinus carpio* and other species attractive for sport fishing. Birds shown in the diet were also connected to water and marsh habitats. The Northern goshawk, on the contrary, hunted mainly in forests or close to human settlements [8].

The two species whose diets overlapped to the highest extent were the common buzzard and common kestrel, and their diet composition overlapped also with the long-eared owl. Similar results were obtained by other researchers [27,29]. The common buzzard and common kestrel were also the species with one of the most diversified diets, yet approximately one-third of prey items caught by both species were field rodents. This suggested they used the same hunting areas, i.e., open fields. Spatial space use partitioning likely occurred in this case. In our study area, common kestrels bred outside the forest, on church towers, and on grain silos. Thus, they hunted mainly in open fields, far from forests. On the contrary, most of the common buzzard nests were located in small forests (however, note that in recent decades, part of the breeding pairs nested in tree groups and tree alleys [6]), and common buzzards could easily reach open areas outside these forests to hunt. It may be assumed that open fields close to the wood edge were penetrated the most intensively [50]. The common buzzard was the most numerous avian predator, nesting in all forest complexes, while there were only three to four pairs of common kestrels breeding each year over the whole study area. Another food component that appeared at high numbers in diets of both species was soricomorphs. We may assume that this resulted from the low accessibility of small rodents (i.e., low rodent density) in 2011; thus, soricomorphs became an alternative food source [6]. Common buzzards, being a food opportunist [51], react to changes in rodent availability by switching to other food categories [52,53,54,55,56]; the same can be observed for kestrels [57].

The species that showed high niche overlap with the common buzzard and common kestrel was the long-eared owl. Out of all species under study, the long-eared owl preyed on field rodents most often, i.e., they accounted for more than 80% of all consumed prey items. The long-eared owl is known to be a food specialist (thus, it had the narrowest food niche of all the species in question), preying mostly on rodents (mainly voles) that inhabit open areas [58]. Even with generally low numbers of common voles in our study area and a further drop in their numbers in recent decades [6,50], they still were staple food for the long-eared owl, while other avian predators (e.g., the common buzzard) switched to other field rodent species [6].

The last species that was added in the group with the common kestrel, common buzzard, and long-eared owl was the tawny owl. This owl also preyed on field rodents, however less intensively than the three aforementioned species. On the contrary, forest rodents were more important for the tawny owl. The tawny owl is a food opportunist (the food niche breadth of the species reached the second highest value), and its diet changes with the prey availability and human presence level, i.e., rural to urban gradient [13,14,38]; therefore, it is able to avoid food competition with other co-occurring avian predators. Although rodents are an important food component for the tawny owl (which affects its breeding success [33]) as an opportunistic feeder [38,39,40,41,42,43,44,45,46,47,48,49,50,51,52,53,54,55,56,57,58,59], the tawny owl is an effective predator of both forest rodents (mainly the yellow-necked mouse), and those of field habitats (voles and field-striped mice), as well as birds [60]. This plasticity seems to be the key to success as, despite changes in environmental conditions, the species remained stable in number in our study area [33].

Birds were found mostly in the diets of the Northern goshawk and sparrowhawk (over 80% of identified prey items) and the diets of those two species overlapped considerably (the index value being 0.54). Medium-sized and big birds, like pigeons *Columba* spp. and poultry, were the most numerous in the diet of the Northern goshawk [8], while small birds were the most numerous in the diet of the sparrowhawk. Such niche partitioning is typical for the two species, allowing for their coexistence in the same area [61]. The other species, who preyed on small birds (32% of prey items) was the hobby, which was in line with the findings of other researchers [28,62]. The food niche breadth of the hobby was rather low (2.0), and it overlapped to a large extent only with the food niche of the sparrowhawk (0.54). Yet, this hawk also preyed heavily on insects (which accounted for over 60% of all prey items), a food source used far less intensively by the other avian predators in the study area.

## 5. Conclusions

Our research was carried out in a highly transformed landscape, with small woods surrounded by open, agricultural areas. The avian predators assemblage reached very high density. Avian predators bred mostly in the forest. Yet, they searched for food in various habitats, i.e., in the woods, outside—in agricultural areas or at fishponds, they also made use of food of anthropogenic origin (i.e., poultry or domestic pigeons). Our results confirmed that the food niches of species coexisting in the same area were considerably separate. In this case, thanks to high habitat heterogeneity, birds could hunt on various prey species. Space use partitioning also occurred as birds searched for prey in different habitats [15,30] or probably in different patches of the same habitats. Detailed mechanism for space use partitioning between the species could be a topic of future studies involving, e.g., satellite telemetry technics.

## Figures and Tables

**Table 1:** The mean number of breeding pairs of avian predators in the breeding season in the area of study (central Poland, vicinity of Rogów village, Experimental Forest Station of Warsaw University of Life Sciences). * Species that were included in the food niche overlap analysis presented in this paper are marked with an asterisk. The source of the abundance data is given in brackets.

Species	N	(Min–Max)	Study Years [Data Source]
*Buteo buteo* *	37.2	36–39	2011–2018 [6]
*Accipiter gentilis* *	8.0	6–11	2011–2018 [8]
*Accipiter nisus* *	20.1	18–22	2011–2017 [34]
*Haliaeetus albicilla* *	1.4	1–2	2008–2018 [35]
*Falco subbuteo* *	3.5	3–5	2011–2015 [35]
*Falco tinnunculus* *	3.5	3–4	2011–2015 [36]
*Circus aeruginosus*	3.0	3–3	2011–2015 [36]
*Circus pygargus*	2.0	2–2	2011–2015 [36]
*Pernis apivorus*	3.0	3–3	2011–2015 [35]
*Strix aluco* *	27.0	26–29	2004–2018 [32,33]
*Asio outs* *	16.0	11–21	2004–2008 [32]
*Corvus corax*	7.5	6–13	2011–2018 [37]
Total (12 species)	133.2		

**Table 2:** The diet composition (% of each type of prey) and niche breadth of avian predators in the breeding season in the area of study (central Poland, vicinity of Rogów village, Experimental Forest Station of Warsaw University of Life Sciences), based on pellet analysis and prey remains collection. The food niche breadth was calculated according to the Simpson formula.

Prey	*Buteo buteo*	*Accipiter gentilis*	*Accipiter nisus*	*Haliaeetus albicilla*	*Falco subbuteo*	*Falco tinnunculus*	*Strix aluco*	*Asio otus*
Soricomorphs	19.1	0.2	0.4	0.3	0.4	12.4	3.3	0.9
Forest rodents	3.5	2.6	4.0	0	1.8	0.4	33.2	2.2
Field rodents	30.3	1.0	1.2	0.6	1.5	31.1	21.5	81.5
Other mammals	19.7	7.7	2.5	12.2	1.4	27.5	4.9	2.9
Big birds	1.9	33.6	2.0	42.5	0	0	0.2	0
Medium birds	11.9	43.1	28.5	4.9	0.4	2.0	3.2	0.3
Small birds	10.1	8.5	51.0	0	31.6	17.4	10.1	5.9
Amphibians	0.4	0.1	0.1	0.6	0	0	2.2	0.1
Reptails	1.3	0.1	0.1	0.9	0.4	1.0	0.4	0.1
Fishes	0	0	0	33.3	0	0	0	0
Insects	1.9	3.1	10.3	4.6	62.6	8.2	20.9	6.0
N of prey items	5103	1065	930	327	281	695	1601	917
Niche breadth	5.2	3.2	2.8	3.2	2.0	4.4	4.6	1.5
Study years	2011–2018	2011–2018	2011–2017	2008–2018	2011–2018	2011–2018	2003–2018	2004–2018
Source of data	[6]	[8]	[34]	This study (Table A1)	This study (Table A3)	This study (Table A2)	[38], unpubl. data 2015–2018	[39,40], unpubl. data 2015–2018

**Table 3:** The food niche overlap of avian predators in the breeding season in the area of study (central Poland, vicinity of Rogów village, Experimental Forest Station of Warsaw University of Life Sciences), based on pellet analysis and prey remains collection and calculated with a Pianka formula [48].

Species	*B. buteo*	*A. gentilis*	*A. nisus*	*H. albicilla*	*F. subbuteo*	*F. tinnunculus*	*S. aluco*	*A. otus*
***B. buteo***		0.34	0.36	0.16	0.15	0.93	0.54	0.61
***A. gentilis***			0.54	0.56	0.12	0.19	0.17	0.04
***A. nisus***				0.09	0.54	0.39	0.35	0.09
***H. albicilla***					0.08	0.15	0.07	0.02
***F. subbuteo***						0.32	0.49	0.12
***F. tinnunculus***							0.55	0.62
***S. aluco***								0.46
**Mean**	0.44	0.28	0.34	0.16	0.26	0.45	0.38	0.28

## Data Availability

All data is presented in the paper (new data as tables or  Appendix A) or are given in the cited papers (for which references are given).

## Appendix A

**Table A1:** The diet composition of the white-tailed eagle (*Haliaeetus albicilla*) in the breeding seasons 2008-2018 in the area of study (central Poland, vicinity of Rogów village, Experimental Forest Station of Warsaw University of Life Sciences), based on pellet analysis and prey remains collection. N – number of prey items.

Prey	N	%
	of Prey	
*Anas platyrhynchos*	24	7.3
*Aythya fuligula*	5	1.5
*Aythya ferina*	3	0.9
Anatidae	9	2.8
*Anser* sp.	2	0.6
*Fulica atra*	5	1.5
*Grus grus* juv.	1	0.3
*Gallinula chloropus*	2	0.6
*Pediceps cristatus*	2	0.6
*Ardea cinerea*	2	0.6
*Ardea alba*	3	0.9
*Ciconia ciconia*	2	0.6
*Phalacrocorax carbo*	1	0.3
*Chroicocephalus ridibundus*	2	0.6
*Cygnus olor* juv.	2	0.6
*Columba palumbus*	5	1.5
*Garrulus glandarius*	3	0.9
*Pica pica*	2	0.6
*Buteo buteo*	4	1.2
*Phasianus colchicus*	2	0.6
*Accipiter* sp.	1	0.3
*Dryocopus martius*	1	0.3
*Columba livia*	1	0.3
*Gallus domesticus*	7	2.1
Medium bird indet.	16	4.9
Big bird indet.	48	14.7
*Talpa europaea*	1	0.3
*Microtus* sp.	2	0.6
*Castor fiber*	1	0.3
*Rattus norvegicus*	2	0.6
*Ondatra zibethicus*	1	0.3
*Lepus europaeus*	1	0.3
*Canis familiaris*	13	4.0
*Vulpes vulpes*	2	0.6
*Felis catus*	9	2.8
*Neovison vison*	1	0.3
*Lutra lutra* juv.	1	0.3
*Sus domestica*	4	1.2
*Sus scrofa*	2	0.6
*Capreolus capreolus*	3	0.9
*Cyprinus carpio*	61	1.,7
*Ctenopharyngodon idella*	3	0.9
*Carassius gibelio*	5	1.5
*Hypophthalmichthys* sp.	2	0.6
*Tinca tinca*	1	0.3
Cyprinidae indet.	31	9.5
*Perca fluviatilis*	2	0.6
*Rutilis rutilis*	1	0.3
*Abramis brama*	1	0.3
*Esox lucius*	1	0.3
*Acipenser* sp.	1	0.3
*Natrix natrix*	3	0.9
Ranidae	2	0.6
Insecta	15	4.6
Total	327	100.0

**Table A2:** The diet composition of kestrel (*Falco tinnunculus*) in the breeding seasons 2011–2018 in the area of study (central Poland, vicinity of Rogów village, Experimental Forest Station of Warsaw University of Life Sciences), based on pellet analysis and prey remains collection. N – number of prey items.

Prey	N	%
	of Prey	
*Microtus arvalis*	39	5.6
*Microtus economus*	9	1.3
*Microtus* spp.	76	10.9
*Myodes glareolus*	1	0.1
*Apodemus agrarius*	42	6.0
*Apodemus sylvaticus*	7	1.0
*Apodemus flavicollis*	2	0.3
*Apodemus* spp.	57	8.2
*Mus musculus*	39	5.6
*Micromys minutus*	4	0.6
Rodentia indet.	134	19.3
*Talpa europaea*	9	1.3
*Sorex araneus*	55	7.9
*Sores minutus*	17	2.4
*Sorex* spp.	5	0.7
Small bird indet.	121	17.4
Medium bird indet.	14	2.0
Lacertidae	7	1.0
Insecta	57	8.2
Total	695	100.0

**Table A3:** The diet composition of hobby (*Falco subbuteo*) in the breeding seasons 2011–2018 in the area of study (central Poland, vicinity of Rogów village, Experimental Forest Station of Warsaw University of Life Sciences), based on pellet analysis and prey remains collection. N – number of prey items.

Prey	N	%
	of Prey Items	
Hirundinidae	12	4.3
*Apus apus*	4	1.4
*Dendrocopos major*	2	0.7
*Alauda arvensis*	2	0.7
*Motacilla alba*	2	0.7
*Erithracus rubecula*	7	2.5
*Turdus* sp.	1	0.4
*Sylvia* spp.	4	1.4
Paridae	12	4.3
*Sitta europaea*	3	1.1
*Fringilla coelebs*	7	2.5
*Phylloscopus* spp.	5	1.8
Small bird indet.	29	1.3
*Sorex araneus*	1	0.4
*Myodes glareolus*	5	1.8
*Apodemus agrarius*	2	0.7
*Microtus* sp.	1	0.4
*Micromys minutus*	1	0.4
Rodentia indet.	4	1.4
Lacertidae	1	0.4
Insecta	176	62.6
Total	281	100.0
